# Aqueous Extract of *Nypa fruticans* Wurmb. Vinegar Alleviates Postprandial Hyperglycemia in Normoglycemic Rats †

**DOI:** 10.3390/nu7085320

**Published:** 2015-08-20

**Authors:** Nor Adlin Yusoff, Mariam Ahmad, Bassel Al-Hindi, Tri Widyawati, Mun Fei Yam, Roziahanim Mahmud, Khairul Niza Abdul Razak, Mohd Zaini Asmawi

**Affiliations:** 1 School of Pharmaceutical Sciences, Universiti Sains Malaysia, Penang 11800, Malaysia; E-Mails: mariam@usm.my (M.A.); basselalhindi@yahoo.com (B.H.); tw_rozan@yahoo.com (T.W.); yammunfei@yahoo.com (M.F.Y.); rozi@usm.my (R.M.); niza@usm.my (K.N.A.R.); amzaini@usm.my (M.Z.A.); 2 Integrative Medicine Cluster, Advanced Medical and Dental Institute, Universiti Sains Malaysia, Penang 13200, Malaysia; 3 Pharmacology & Therapeutic Department, Medical Faculty, University of Sumatera Utara, Medan 20155, Indonesia

**Keywords:** *Nypa fruticans* Wurmb, vinegar, postprandial hyperglycemia, oral glucose tolerance test, alpha glucosidase, acarbose

## Abstract

*Nypa fruticans* Wurmb. vinegar, commonly known as nipa palm vinegar (NPV) has been used as a folklore medicine among the Malay community to treat diabetes. Early work has shown that aqueous extract (AE) of NPV exerts a potent antihyperglycemic effect. Thus, this study is conducted to evaluate the effect of AE on postprandial hyperglycemia in an attempt to understand its mechanism of antidiabetic action. AE were tested via *in vitro* intestinal glucose absorption, *in vivo* carbohydrate tolerance tests and spectrophotometric enzyme inhibition assays. One mg/mL of AE showed a comparable outcome to the use of phloridzin (1 mM) *in vitro* as it delayed glucose absorption through isolated rat jejunum more effectively than acarbose (1 mg/mL). Further *in vivo* confirmatory tests showed AE (500 mg/kg) to cause a significant suppression in postprandial hyperglycemia 30 min following respective glucose (2 g/kg), sucrose (4 g/kg) and starch (3 g/kg) loadings in normal rats, compared to the control group. Conversely, in spectrophotometric enzymatic assays, AE showed rather a weak inhibitory activity against both α-glucosidase and α-amylase when compared with acarbose. The findings suggested that NPV exerts its anti-diabetic effect by delaying carbohydrate absorption from the small intestine through selective inhibition of intestinal glucose transporters, therefore suppressing postprandial hyperglycemia.

## 1. Introduction

Diabetes mellitus (DM) is a chronic metabolic disorder caused by impairment of insulin production by pancreatic β cells and/or defects in insulin action [1]. Such abnormalities lead to chronic hyperglycemia, which, in turn, deranges the metabolism of carbohydrates, proteins and fats, and results in several macro- and micro-vascular complications. Postprandial hyperglycemia is one of the earliest detectable abnormalities signaling type 2 diabetes mellitus [2]. Relevant clinical studies suggested that it was an important factor contributing to the development of diabetes complications [3,4]. Hence, to prevent or slow the manifestation of diabetes-related complication, good management of postprandial hyperglycemia is critical early in the treatment of diabetes mellitus. One way to achieve controlled postprandial blood glucose levels is to slow down glucose absorption in the intestines by inhibition of the action of certain carbohydrate-hydrolyzing enzymes, namely pancreatic α-amylase, and intestinal α-glucosidase and glucose transporters like SGLT 1 and GLUT 2 [5].

In the 1990s, α-glucosidase inhibitors such as acarbose, voglibose and miglitol were approved as oral drugs for the treatment of diabetes mellitus. Currently, they are widely used, either alone or in combination with diet and insulin therapy, in patients with type 1 and type 2 DM [6,7]. Furthermore, canagliflozin and dapagliflozin were among the earliest glucose transporter SGLT2 inhibitors approved by the Food and Drug Administration (FDA) for the purpose of achieving better glycemic control in adults with type 2 diabetes mellitus. Aside from these drugs, many other SGLT inhibitors are being developed and are currently undergoing clinical trials [8].

*Nypa fruticans* Wurmb. vinegar, locally known as nipa palm vinegar (NPV) is a traditional preparation produced by the fermentation of nira, an alcoholic beverage of nipa palm (*Nypa fruticans* Wurmb.) sap. It is commonly consumed throughout East Asia [9]. Added to drinking water, NPV is taken before meals and at bedtime. It has been shown that the ingestion of vinegar reduces postprandial hyperglycemia in type 2 diabetic patients receiving meals of moderate glycaemic index [10]. In our previous studies, chronic administration of NPV aqueous extract at a dose of 1000 mg/kg caused a significant blood glucose lowering effect and improved the level of insulin of diabetic rats [11]. Hence, the effects of the aqueous extract on intestinal glucose absorption and postprandial hyperglycemia were further studied in an attempt to understand the mechanisms of its blood glucose lowering activity.

## 2. Experimental Section

### 2.1. Chemicals

α-glucosidase, porcine α-amylase and d(+)-glucose were purchased from Sigma Aldrich Chemical (St. Louis, MO, USA). Sucrose and starch were supplied by Sigma, Life Science (Waltham, MA, USA). Phloridzin and acarbose (Glucobay^®^) were respectively obtained from Sigma-Aldrich (St. Louis, MO, USA) and Bayer AG (Wuppertal, Germany). All reagents were of analytical grade.

### 2.2. Plant Preparation and Extraction

NPV used in the study was supplied by a local producer from Titi Bakong, Yan, Kedah, Malaysia (5°48′9.42″ N, 100°22′35.32″ E). Several parts of nipa palm were authenticated by Rahmad Zakaria at the Herbarium Unit, School of Biological Sciences, Universiti Sains Malaysia and the voucher specimens were deposited at the same unit with the voucher number of USM. Herbarium 11541. NPV was made by the fermentation of nipa palm sap “nira”. In brief, nira was stored in a polyethylene barrel for 44 days at room temperature to allow a slow and natural fermentation process of alcohol and acetic acid contents. Fully fermented product, NPV with a pH around 3.2 was then filtered, poured into bottles and made ready for consumption.

NPV was extracted using the liquid–liquid extraction approach described by Qiu *et al.* [12] with minor modifications. The vinegar (500 mL) was concentrated to a final volume of 250 mL at 37 °C using a vacuum rotary evaporator (Labortechnik, AG CH-9230, Postfach, Flawil, Switzerland). The concentrated vinegar was first extracted with ethyl acetate in a ratio of 1:1 using a separatory funnel. The upper layer of ethyl acetate was then separated, and the residue, the aqueous layer, was collected. The liquid-liquid partitioning step between ethyl acetate and aqueous NPV was repeated again for three batches. The aqueous layer containing potential bioactive was collected, pooled, concentrated under reduced pressure at 40 °C using a vacuum rotary evaporator to remove traces of organic solvent, and lyophilized using a freeze-drier (Labconco Corporation, Kansas, MO, USA) to yield the aqueous extract (yield 85%). Finally, the aqueous extract (AE) was stored in the freezer at −4 °C until further use.

### 2.3. Animals

Healthy adult male Sprague-Dawley rats (200 to 250 g) were obtained from the Animal Research and Service Centre (ARASC), USM. In order to acclimatize to the laboratory environment, prior to experimentation, the animals were housed in a well-ventilated animal transit room and fed a standard diet and water *ad libitum* for a period of seven days. The experiment procedure was approved by the Animal Ethics Committe, Universiti Sains Malaysia (Approval number: USM/Animal Ethics Approval/2011/(71)(326)).

### 2.4. Intestinal Glucose Absorption by the Everted Sac Technique

AE effect on glucose absorption via isolated rat jejunal sacs was studied according to the method of Wilson & Wiseman [13], as described by Hassan *et al.* [14]. Following an overnight fast, a male Sprague Dawley rat (250–300 g) was sacrificed and the abdominal wall was dissected. The jejunum was isolated from the small intestine (20 to 50 cm from the pylorus), placed in a Tyrode buffer solution (342 mM NaCl, 6.7 mM KCl, 5.9 mM CaCl_2_·2H_2_O, 5.3 mM MgCl_2_, 59.5 mM NaHCO_3_, 2.08 mM NaH_2_PO_2_ and 5.5 mM glucose) and aerated with carbogen (95% O_2_ and 5% CO_2_). The jejunum was everted and cut into 5-cm segments. Each segment, filled with 1 mL of the Tyrode solution, was formed into a sac by tying both of its ends with cotton threads. Each sac was further incubated for 90 min at 37 °C in a test tube containing a total of 15 mL of the Tyrode solution. Added to the tubes, the test substances were comprised of AE (1 mg/mL), acarbose (1 mg/mL) and phloridzin (0.001 M), respectively. The tubes containing the Tyrode solution alone served as the negative control. The initial and final glucose concentrations following the incubation period were measured using a chemistry analyser, Stat Fax 1937 (Awareness Technology Inc., Palm City, FL, USA); and the intestine glucose absorption could be calculated as follows:

Amount of glucose absorbed (mg/g tissue weight) = ((G_before_ − G_after_)/W_intestine_) (1)

where G_before_ and G_after_ represented glucose concentrations (mg/dL) before and after incubation, respectively; whereas W_intestine_ represented the weight of the intestinal segment (g).

### 2.5. Oral Glucose Tolerance Test (OGTT)

As detailed by Ali *et al.* [15], overnight-fasted non-diabetic rats were divided into five groups of six rats each and received the treatment orally: Group 1 was given distilled water (10 mL/kg). Groups 2 and 3 were treated with acarbose (10 mg/kg) and phloridzin (200 mg/kg), respectively, to serve as the positive controls. Groups 4 and 5 were treated with AE at the respective doses of 500 mg/kg and 250 mg/kg. Ten minutes after a single oral administration as above, the rats were challenged orally with glucose at a dose of 2 g/kg B.W. Blood samples were taken from the tail tip at 0 (before treatment), 30, 60 and 120 min after the glucose challenge. Blood glucose levels were determined using a glucometer (Accu-Check, Roche, Mexico City, Mexico) utilizing the glucose oxidase-peroxidase method. Areas under the curve (AUC) were calculated using the trapezoidal method.

### 2.6. Oral Sucrose Tolerance Test (OSucTT)

The procedure mentioned in Section 2.5 was applied to a similar set of animals. However, sucrose, at a dose of 4 g/kg B.W. was administered in place of glucose.

### 2.7. Oral Starch Tolerance Test (OSTT)

The same procedure as in Section 2.5 was applied. Starch was administered in place of glucose and given at a dose of 3 g/kg B.W.

### 2.8. In Vitro Enzymatic Inhibition Study: α-Glucosidase

AE α-glucosidase inhibition activity was assessed spectrophotometrically as previously described by Kwon *et al.* [16] with slight modifications. Briefly, 50-μL aliquots of AE at different concentrations (6.25–100.00 mg/mL) were mixed with 100 μL of a 0.1 M phosphate buffer (pH 7.0) containing 0.5 U/mL of α-glucosidase to be incubated in a 96-well plate at 37 °C for 10 min. The control contained a similar aliquot of the 0.1 M phosphate buffer solution instead of the extract. After incubation, 50 μL of a solution containing 5 mM *p*-nitrophenyl-α-d-glucopyranoside (pNPG) in a 0.1 M phosphate buffer (pH 7.0) were added to each well. The reaction mixture was further incubated at 37 °C for 5 min. The absorbances at before and after the incubation period were recorded using a microplate reader (Power Wave × 340, BioTek^®^ Instruments Inc., Winooski, VT, USA) at λ_max_ 490 nm. Acarbose was served as the standard reference and treated similarly to the sample. The percentage of inhibition of the activity of α-glucosidase was calculated using the following equation:

α-glucosidase inhibitory activity (%) = [(A_control_ − A_sample_)/A_control_] × 100 (2)

where A_control_ represented the absorbance of the control. A_sample_ represented the absorbance of the sample. The concentration of the sample/acarbose required to inhibit 50% of the α-glucosidase activity (IC_50_) was determined by the linear regression analysis.

### 2.9. In Vitro Enzymatic Inhibition Study: α-Amylase

AE α–amylase inhibition activity was studied according to the method described by Kwon *et al.* [16]. 500 μL of AE and acarbose (standard α-amylase inhibitor) were each mixed with 500 μL of a 20 mM sodium phosphate buffer (pH 6.9 with 6 mM sodium chloride) containing 0.5 mg/mL of α-amylase. The control mixture contained a similar volume of the 20 mM sodium phosphate buffer in place of the extract. The resulting solutions were incubated at 25 °C for 10 min. Then, 500 μL of a 1% starch solution, prepared in 20 mM sodium phosphate buffer (pH 6.9 with 0.006 M sodium chloride), were added. The final mixtures were incubated at 25 °C for 10 min as well; and the reaction was stopped by the addition of 1.0 mL of dinitrosalicylic acid (DNSA), a colour reagent. Then, reaction tubes were incubated in a boiling water bath (Buchi, B-491, Postfach, Flawil, Switzerland) for 5 min. Cooled down to room temperature, the solutions were diluted with 10 mL of distilled water. Lastly, using a UV-250 UV–Visible spectrophotometer (Shimadzu Corporation, Tokyo, Japan), absorbance values were obtained at λ_max_ 540 nm; and the inhibitory activities of AE and acarbose were calculated as follows:

α-amylase inhibitory activity (%) = [(A_control_ − A_sample_)/A_control_] × 100 (3)

where A_control_ denoted the absorbance of the control. A_sample_ denoted the absorbance of the experimental sample. The inhibitory activities of the samples against α-amylase were expressed as the percentage of inhibition, and IC_50_ values were reported.

### 2.10. Statistical Analysis

Data was expressed as the mean ± standard error of the mean (SEM). Statistical significance was assessed using one-way analysis of variance (ANOVA) followed by Dunnett’s Test or Tukey HSD (honest significant difference) as the *post hoc* tests. *p* values less than 0.05 indicated statistical significance.

## 3. Results

### 3.1. AE Attenuates Intestinal Glucose Absorption

**Figure 1:**
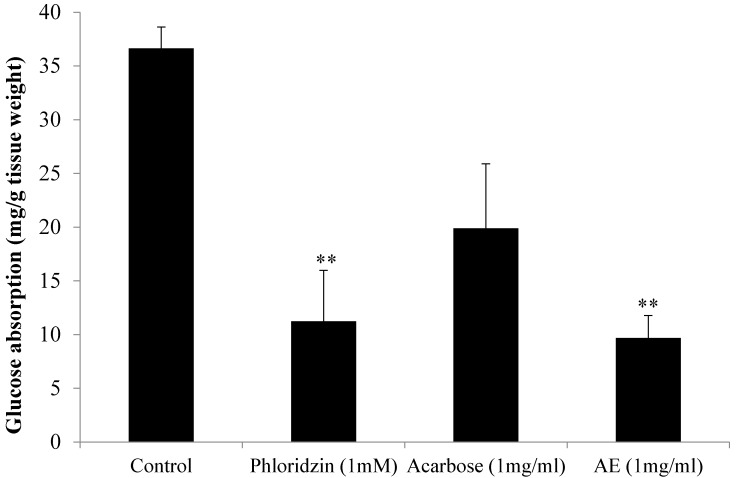
Effect of control, acarbose, phloridzin and aqueous extract (AE) of NPV on intestinal glucose absorption by everted sac technique. Data is represented as means ± S.E.M. (*n* = 6). ** *p* < 0.01 *vs.* the control group.

Figure 1 depicts the *in vitro* effects of AE, acarbose and phloridzin on intestinal glucose absorption, as investigated using the everted jejunal sac technique. Acarbose, an α-glucosidase inhibitor, and phloridzin, a SGLT inhibitor, were tested for comparison purposes. As shown below, in the absence of test substances, the amount of absorbed glucose was 36.63 mg/g tissue weight. The presence of phloridzin, however, caused a significantly reduced glucose absorption rate (*p <* 0.01) of 11.23 mg/g tissue weight (an inhibition of 69.34%) in comparison with the control. AE recorded a similar promising outcome as the amount of glucose absorbed significantly decreased to 9.69 mg/g tissue weight (*p <* 0.01) when compared to the control (a 73.54% reduction in absorption). On the other hand, acarbose caused a 45.67% reduction in glucose absorption, as the absorption rate was 19.9 mg/g tissue weight only. However, although the reduction percentage was quite high, it was not significant compared with the control (*p* < 0.056).

### 3.2. Oral Glucose Tolerance Test (OGTT) in Normal Rats

**Figure 2:**
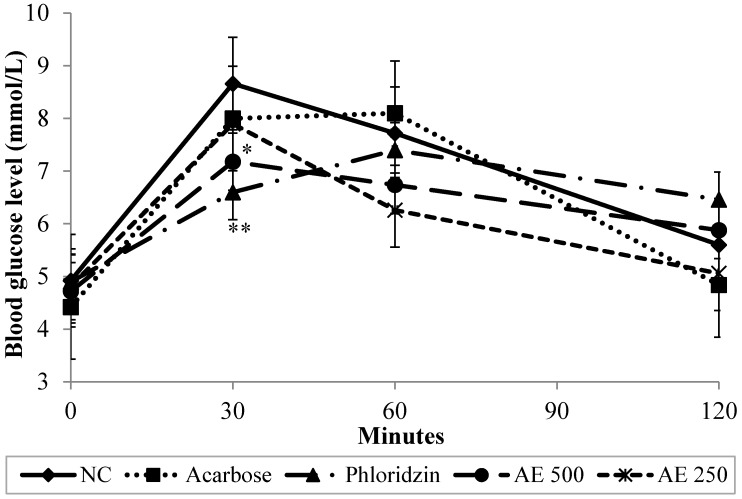
Effect of normal control (NC), acarbose, phloridzin and aqueous extract (AE) of NPV on postprandial blood glucose levels in oral glucose tolerance test. Data is expressed as means ± S.E.M. (*n* = 6). * *p* < 0.05, ** *p* < 0.01 *vs.* the NC group.

To confirm the intestinal inhibitory effect of AE, an *in vivo* glucose challenge test was carried out. Figure 2 showed that in the normal control group (NC), the postprandial hyperglycemia levels caused by 2 g/kg of glucose loading reached 8.66 mmol/L 30 min after glucose administration. 60 min afterwards, they decreased to 7.72 mmol/L and continued to reach an average of 5.6 mmol/L 120 min post-loading. However, given at a dose of 500 mg/kg, compared with the control group, AE caused the elevation of the blood glucose levels to be significantly suppressed (*p <* 0.01) at minute 30. Likewise, phloridzin caused a significant (*p <* 0.01) suppression in the rise of the blood glucose levels 30 min after glucose loading. Hence, it can be concluded that a dose of 500 mg/kg of AE exhibited a comparable effect to phloridzin at a dose of 200 mg/kg in suppressing postprandial hyperglycemia. However, as the blood glucose levels decreased naturally in the NC group, no statistical significance could be observed between the NC, and AE and phloridzin groups later on. On the other hand, a dose of 10 mg/kg of acarbose did not significantly suppress the rise in blood glucose levels after glucose loading throughout the course of the study. As seen in Table 1, the area under the curve (AUC) of glucose tolerance for the AE-treated groups (AE 500; 765.9 ± 62.3 mmol·min/L and AE 250; 742.8 ± 46.4 mmol·min/L) was significantly lower than that of the NC group (849.0 ± 59.5 mmol·min/L) and comparable to those of both phloridzin (798.3 ± 75.6 mmol·min/L) and acarbose (816.0 ± 60.2 mmol·min/L). AUC values corroborated with the recorded blood glucose levels.

**Table 1:** Area under the curve (AUC) of postprandial glucose responses of normoglycemic rats in oral glucose tolerance test (OGTT), oral sucrose tolerance test (OSucTT) and oral starch tolerance test (OSTT).

Group ^1^	AUC (mmol·min/L)		
	OGTT	OSucTT	OSTT
NC	851.7 ± 59.5 ^b^	855.0 ± 58.8 ^c^	840.6 ± 55.5 ^c^
Acarbose	816.0 ± 60.2 ^ab^	668.7 ± 58.9 ^a^	623.7 ± 45.8 ^a^
Phloridzin	798.3 ± 75.6 ^ab^	676.2 ± 63.9 ^a^	685.5 ± 62.0 ^ab^
AE 500	756.3 ± 62.3 ^a^	737.4 ± 58.0 ^b^	756.6 ± 58.6 ^bc^
AE 250	742.8 ± 46.4 ^a^	776.7 ± 62.6 ^b^	768.9 ± 56.1 ^bc^

^1^ NC (10 mL/kg of distilled water), acarbose (10 mg/kg), phloridzin (200 mg/kg), and AE (500 mg/kg). Treatments were co-administered orally and respectively with glucose (2 g/kg), sucrose (4 g/kg), and starch (3 g/kg) in OGTT, OSucTT and OSTT tests. Data is expressed as means ± S.E.M. of six rats. Means with different letters of the alphabet within a column are significantly different at *p* < 0.05, as analyzed using Tukey HSD (honest significant difference) as a *post hoc* test.

### 3.3. Oral Sucrose Tolerance Test (OSucTT) in Normal Rats

**Figure 3:**
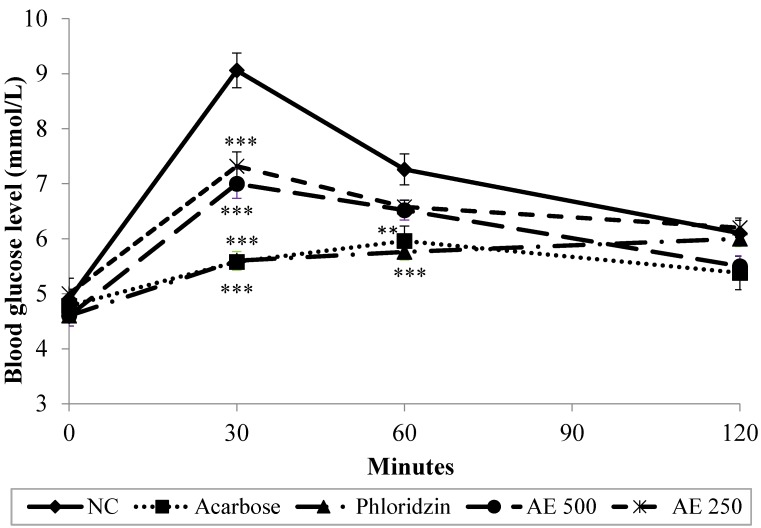
Effect of normal control (NC), acarbose, phloridzin and aqueous extract (AE) of NPV on postprandial blood glucose levels in oral sucrose tolerance test. Data is expressed as means ± S.E.M. (*n* = 6). ** *p* < 0.01, and *** *p* < 0.001 *vs.* the NC group.

Results depicted in Figure 3 show that, in the NC group, the blood glucose levels of the sucrose-loaded rats increased from 4.9 to 9.1 mmol/L 30 min after the oral administration of sucrose (4 g/kg), and then it decreased to 7.3 mmol/L and 6.1 mmol/L 60 and 120 min post-loading, respectively. As observed in OGTT, a dose of 500 mg/kg of AE significantly (*p* < 0.001) attenuated the elevated blood glucose levels 30 min after sucrose loading, with the percentage of blood glucose increment being 34.3% only, as compared with the initial glucose levels. Similar suppression pattern was observed in AE-treated group at a dose of 250 mg/kg. With further observation, an insignificant diminution in blood glucose levels was recorded as the levels decreased to 6.5 mmol/L and 5.5 mmol/L respectively 60 min and 120 min post-loading. On the other hand, as compared to the control group, 200 mg/kg and 10 mg/kg of phloridzin and acarbose, respectively, significantly suppressed postprandial hyperglycemia until 60 min after sucrose-loading. Furthermore, acarbose continued to significantly suppress glucose levels until the end of the study. AUC readings reflected the recorded blood glucose levels, as lower AUC values indicated lower glucose concentrations in the blood, suggesting a stronger inhibitory effect of intestinal glucose absorption for the test substance. Hence, AUC trend was as follows: NC > AE 250 > AE 500 > Phloridzin > Acarbose (Table 1).

### 3.4. Oral Starch Tolerance Test (OSTT) in Normal Rats

**Figure 4:**
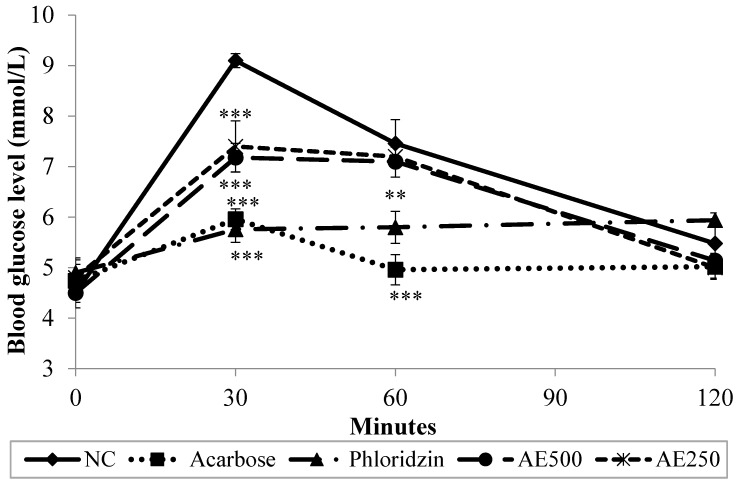
Effect of normal control (NC), acarbose, phloridzin and aqueous extract (AE) of NPV on postprandial glucose levels in oral starch tolerance test. Data is expressed as means ± S.E.M. (*n* = 6). ** *p* < 0.01, and *** *p* < 0.001 *vs.* the NC group.

Figure 4 depicts the changes in postprandial blood glucose levels as observed in oral starch tolerance test. Thirty min following starch administration, the blood glucose levels of NC rose up to 9.10 mmol/L. Later, at minutes 60 and 120 after starch was administered, the glucose levels decreased to 7.46 mmol/L and then to 5.48 mmol/L, respectively. However, 500 mg/kg of AE significantly (*p* < 0.001) suppressed postprandial hyperglycemia as obsereved 30 min after starch administration, when compared with NC, with not more than a 37.3% rise in blood glucose levels being recorded at that point. Though statistically insignificant, AE blood glucose lowering effect was sustained until the end of the study. Similar observation was recorded in AE 250 group. Likewise, phlorizine, at a dose of 200 mg/kg, caused a comparable significant suppression effect to that of acarbose (10 mg/kg) at minute 30 (*p* < 0.001), as compared to NC. Both phloridzin and acarbose sustained this significant effect until minute 60 (*p* < 0.01 and *p* < 0.001, respectively). Based on AUC values in Table 1, the NC group showed the highest concentrations of blood glucose per min, with the value being 840.6 ± 55.5 mmol·min/L, which was followed by AE 250 and AE 500 with the values of 768.9 ± 56.1 mmol·min/L and 756.6 ± 58.6 mmol·min/L, respectively. Yet, the AUC value of AE-treated groups was not significantly different from that of phloridzine (685.5 ± 62.0 mmol·min/L). Meanwhile, acarbose treatment showed the lowest AUC value (623.7 ± 45.8 mmol·min/L).

### 3.5. In Vitro Inhibitory Effects of AE against α-Glucosidase and α-Amylase

**Figure 5:**
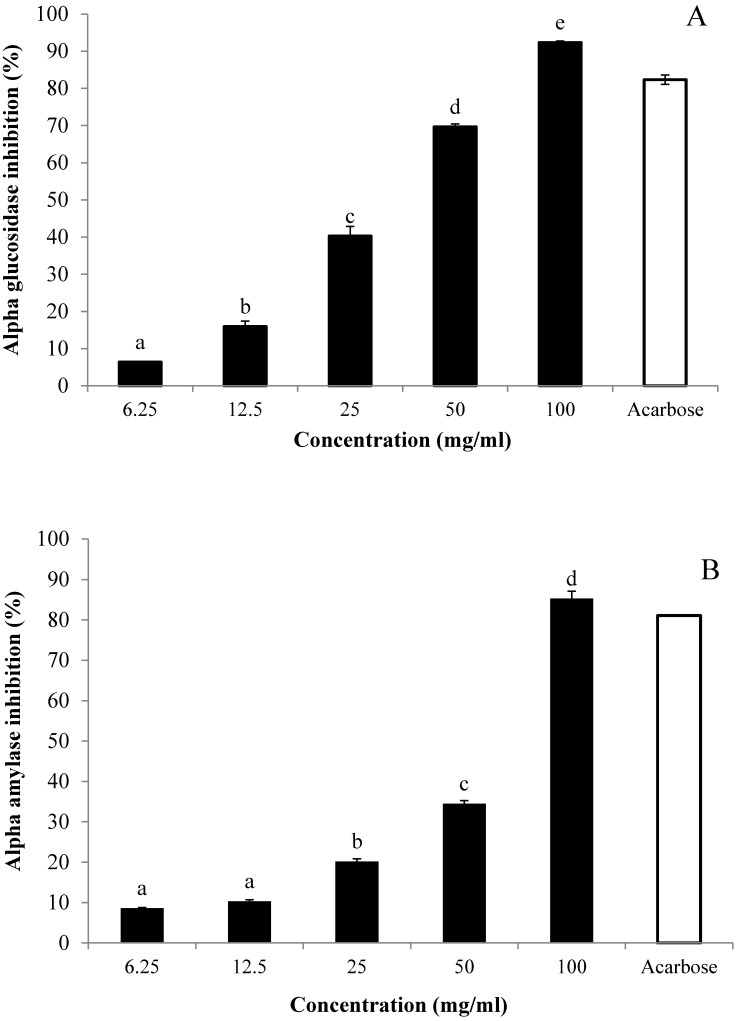
Inhibitory activities of aqueous extract (AE) of NPV on (**A**) α-glucosidase and (**B**) α-amylase. AE inhibitory activities were determined using the respective pNPG and starch as substrates. Acarbose was used as the positive control. Values are expressed as means ± S.E.M. of triplicates. Values with different letters of the alphabet were significantly different at *p* < 0.05, as analyzed using Tukey HSD (honest significant difference) as a *post hoc* test. The final concentration of acarbose was 6.25 mg/mL.

AE inhibitory effects against α-lucosidase and α-mylase were respectively measured using *p*-nitrophenyl-α-d-glucopyranoside (pNPG) and starch as substrates (Figure 5). The inhibition of both enzyme activities was compared with that caused by a standard inhibitor, acarbose. Results indicated that AE inhibited the activity of α-glucosidase in a dose-dependent fashion: The percentages of activity were 0.85%, 6.36%, 15.93%, 40.28%, 69.64%, and 92.33% at the respective concentrations of 3.13, 6.25, 12.50, 25, 50, and 100 mg/mL (Figure 5A). Likewise, the inhibitory effect of AE against α-amylase activity occurred in a dose-dependent manner: The inhibition was intensified gradually as AE concentrations increased from 3.13 to 100 mg/mL (Figure 5B). At the concentration of 100 mg/mL, AE caused the maximum inhibition for both of the enzymes. Yet, with reference to the IC_50_ values of acarbose and AE, findings suggested that AE α-glucosidase and α-amylase inhibitory effects (28.92 ± 0.05 and 60.62 ± 0.32, respectively) were weaker than those of acarbose (1.11 ± 0.02 and 0.45 ± 0.07, respectively).

## 4. Discussion

Postprandial hyperglycemia is characterized by a rapid and great increase in blood glucose levels following a meal [3]. Clinical studies have indicated that postprandial hyperglycemia is an independent and direct risk factor for cardiovascular disease in nondiabetic and diabetic individuals [17]. Management of postprandial hyperglycemia can be achieved by influencing two physiological pathways: (1) The digestion of complex carbohydrates into absorbable monosaccharides by carbohydrate-digestive enzymes; and (2) intestinal absorption of those monosaccharides via intestinal glucose transporters [3]. After ingestion, polysaccharides (e.g., starch) are degraded into oligosaccharides (e.g., sucrose, maltose and lactose) by digestive enzymes in the salivary, along with the pancreatic α-amylase. Then, those oligosaccharides are further broken down into absorbable monosaccharides (e.g., glucose, fructose and galactose) by the α-glucosidase located at the brush border membrane of the intestinal cells, the enterocytes. Once glucose is available in the brush membrane border, enterocytes mediate the expression of a Na^+^/glucose co-transporter, SGLT1 (sodium/glucose-linked transporter 1), and glucose is actively transported into absorptive epithelial cells. To reach the blood circulation, it is then released from those epithelial cells facilitated by a glucose transporter, GLUT2 (glucose transporter 2), which is located in the basolateral membrane [18].

In a diabetic condition, insulin’s ability to stimulate cellular glucose uptake from the blood is compromised [19]. Thus, following a meal rich in carbohydrates, controlling blood glucose at the ideal levels becomes difficult as the body is rendered less capable of responding to released insulin, which results in the accumulation of glucose in the blood, leading to postprandial hyperglycemia. In addition, several studies have shown an increase in the expression of intestinal SGLT1 and the facilitative glucose transporter gene, GLUT 2, in diabetic rats [20,21]—bodily responses which increase the rate of intestinal glucose absorption and further contribute to the onset of postprandial hyperglycemia. The changes in the expression of glucose transporters could be studied by several techniques, namely quantitative real-time PCR analysis, immunohistochemistry and Western blot [22]. For instance, by isolating and quantifying the protein and/or mRNAs of enterocytes that corresponding to SGLT1 and GLUT2 in the intestines of control rats, diabetic rats, and treated-diabetic rats, the expression rates of those key glucose transporters could be compared.

A study done in 2004 showed that consumption of a vinegar preparation (20 g apple cider vinegar, 40 g water and 1 tablespoon saccharin sweetener) as a dietary supplement significantly reduced postprandial blood glucose levels [23]. Moreover, an older study carried on using Caco-2 colon cell culture showed vinegar to decrease the activity of a number of intestinal glucose transporters [24]. In the present study, the effect of the aqueous extract of the vinegar of Malaysia-native *Nypa fruticans* on postprandial hyperglycemia was investigated *in vitro* and *in vivo*. Both phloridzin and acarbose, standard drugs proven to effectively attenuate postprandial glucose levels, were used as the positive controls for comparison purpose. First, *in vitro* intestinal glucose absorption, investigated using isolated and averted rat jejunum, was found to be significantly diminished in the presence of AE or phloridzin. AE’s similitude with phloridzin indicated that it might, as well, act by exerting an inhibitory effect on intestinal glucose transporters, as phloridzin’s principal pharmacological action has been known to be the reduction of the intestinal glucose absorption rate via inhibition of sodium/glucose-linked transporters (SGLTs) located in the mucosal of the small intestine [25]. On the other hand, the results indicated that glucose absorption was not inhibited significantly by acarbose, which was not surprising considering that acarbose has been reported to have no significant inhibitory effect on the absorption of readily available monosaccharides like glucose [26]. The findings of this study, hence, come in support of previous work by Madariaga *et al.* [27], who reported acarbose as a selective α-glucosidase inhibitor which did not affect monosaccaride (namely glucose) transport *in vivo*.

The inhibitory activity of AE was further confirmed *in vivo* using normal rats, which underwent oral glucose, sucrose and starch tolerance tests. Indeed, AE at a dose of 500 mg/kg suppressed the rise in blood glucose levels 30 min after glucose loading (2 g/kg). Phloridzin recorded a similar blood glucose suppression effect as AE. Conversely, however, acarbose, in agreement with the *in vitro* findings using rat isolated jejunum, failed to produce a significant blood glucose lowering effect *in vivo* after glucose loading. In oral sucrose and starch tolerance tests, AE proved to exhibit a similar suppressive activity on elevated blood glucose levels as that seen in OGTT. Additionally, both phloridzin and acarbose significantly attenuated the rise in the blood glucose levels of sucrose and starch loaded rats—an effect which was witnessed as early as in the first 30 min of the 2-h test period. The present *in vivo* test findings suggest that AE probably exerted its antidiabetic effect via suppressing postprandial hyperglycemia. As similar inhibitory effects were seen for AE and phloridzin *in vitro* and *in vivo*, with OGTT, OSucTT and OSTT tests, it can be hypothesized that AE, in part, diminishes glucose absorption through the intestine by inhibition of the activity of intestinal glucose transporters (the same mechanism phloridzin exhibits). It is known that intestinal absorption of glucose is mediated by active transport carried out by the sodium-dependent glucose transporter, SGLT1, and facilitated by the sodium-independent transporter, GLUT2 [28]. As the findings of this study might infer, AE could be ameliorating postprandial hyperglycemia by competing with glucose for the binding site on SGLT1, thus delaying the process of glucose absorption from the diet. Recently, Hassimotto *et al.* [29] proposed that one of the intestinal glucose uptake mechanisms influenced by an anthocyanin probably involved effects on SGLT1. To test this hypothesis, further research is needed in order to pinpoint AE-related effects on the expression and regulation of the facilitative glucose transporters.

On the other hand, further *in vitro* tests have shown AE to exhibit rather a weak inhibitory activity against α-glucosidase and α-amylase (Figure 5), suggesting that AE plays no significant role in the degradation of complex carbohydrates into simple monosaccharides. Intestinal α-glucosidases and pancreatic α-amylase make up a series of carbohydrate metabolizing enzymes, which are involved in the breakdown of complex carbohydrates into absorbable monosaccharides [30]. α-glucosidase and α-amylase inhibitors, like acarbose, can prolong the process of carbohydrate absorption, thus forcing glucose to enter the blood circulation in a latent, rather than a sudden, fashion, eventually attenuating the postprandial rise in blood glucose levels [31]. Considering that AE significantly inhibited intestinal glucose absorption *in vitro*, suppressed the rise of blood glucose levels in OGTT, and possessed weak inhibitory effects on α-glucosidase and α-amylase activities, it could be safely concluded that AE inhibited the transit and absorption of glucose and other monosaccharides in the digestive tract, rather than interfering in the carbohydrate digestion process. Therefore, it could be speculated that the action mechanism of AE is different from that of acarbose.

Previous HPLC analysis of AE revealed a mixture of organic acids, mainly lactic acid and acetic acid at the percentages of 10.35% and 8.68%, respectively, followed by citric acid (6.90%), and succinic acid (5.85%). Malic acid was present at a fair percentage of 2.89%. Acetic acid, found to be one of the major components of AE, has been reported extensively in several studies as having the potential of countering postprandial hyperglycemic and promoting insulinemic responses [32]. Thus, the observed effect of AEs on the postprandial hyperglycemia might be attributable to its acetic acid content.

## 5. Conclusions

Overall, the results of this study demonstrated that an aqueous extract of NPV alleviated postprandial hyperglycemia in normolgycemic rats, at least in part, by delaying the absorption of dietary glucose in the intestine via inhibition of intestinal glucose transporters which are located at the apical side of the enterocytes of the small intestine. The observed suppression of postprandial hyperglycemia by NPV further suggested promising nutraceutical use of the NPV, consumed before and during meals in preventing hyperglycemia.
